# Assessing psychological adjustment and cultural reintegration after military service: development and psychometric evaluation of the post-separation Military-Civilian Adjustment and Reintegration Measure (M-CARM)

**DOI:** 10.1186/s12888-020-02936-y

**Published:** 2020-11-10

**Authors:** Madeline Romaniuk, Gina Fisher, Chloe Kidd, Philip J. Batterham

**Affiliations:** 1 Gallipoli Medical Research Foundation, Greenslopes Private Hospital, Brisbane, Australia; 2 Faculty of Health and Behavioural Sciences, The University of Queensland, Brisbane, Australia; 3 Institute of Resilient Regions, University of Southern Queensland, Toowoomba, Australia; 4 Centre for Mental Health Research, Research School of Population Health, Australian National University, Canberra, Australia

**Keywords:** Reintegration, Adjustment, Transition, Military, Defence, Veteran, Psychometric measure, Scale development

## Abstract

**Background:**

The transition out of military service and subsequent reintegration to civilian life has been established as a period associated with an increased risk of psychological adjustment difficulties, psychiatric disorders and suicide risk, yet no tool exists to measure cultural and psychological adjustment following permanent separation from the military. This study describes the two-phase mixed-methods development and validation of the self-report Military-Civilian Adjustment and Reintegration Measure (M-CARM).

**Methods:**

In Phase I, four focus groups *(n* = 20) and semi-structured one-on-one interviews (*n* = 80) enabled thematic analysis and generation of 53 initial items that were reviewed by an expert multidisciplinary panel (*n* = 12) and piloted for clarity and relevance in an Australian service-veteran sample (*n* = 11). In Phase II, psychometric properties of the 47 items resulting from Phase I were evaluated with online assessment of a convenience sample of transitioned Australian Defence Force veterans (*n* = 725). Analyses included exploratory and confirmatory factor analyses, as well as evaluation of test-retest reliability, internal consistency, and convergent, divergent and discriminant validity.

**Results:**

Exploratory factor analysis on a randomized split-half sample (*n* = 357), resulted in a 21-item, five-factor solution of *Purpose and Connection*, *Help seeking*, *Beliefs about civilians*, *Resentment and regret*, and *Regimentation*, explaining 53.22% of the variance. Confirmatory factor analysis (*n* = 368) verified this factor structure without modification (*χ2* = 304.96, *df* = 160; CFI = .96, TLI = .94, NFI = .91, RMSEA = .05). Strong convergent, divergent and discriminant validity was demonstrated as M-CARM scores significantly correlated with related constructs assessed by standardised clinical measures as well as differentiated groups based on three binary reintegration items, with large effect sizes (*d* = > 1). Strong test-retest reliability for the total score (*n* = 186, *r* = .93) and excellent internal consistency (*n* = 725, *a* = .90) were also found.

**Conclusions:**

Results provide promising evidence the M-CARM is a valid, reliable measure of psychological and cultural reintegration to civilian life, with potential for considerable clinical and research application.

**Supplementary Information:**

The online version contains supplementary material available at 10.1186/s12888-020-02936-y.

## Background

In veteran health literature, reintegration refers to both the dynamic process of resuming a civilian role after deployment or separation, and the outcome of that process [1–3]. Reintegrating successfully is considered vital for veterans’ mental and physical health and social functioning [4–6]. Successful reintegration is holistic, culturally determined [2, 7], and involves complex interactions between the veteran and their environment [2, 6].

While the literature suggests some veterans adjust to civilian society with little difficulty, a significant proportion of veterans experience reintegration challenges [5, 8, 9]. A U.S. investigation found that up to 56% of Iraq and Afghanistan veterans receiving Veteran Affairs medical care reported substantial difficulties reintegrating, with 96% of the sample expressing interest in services to help readjust to civilian life [10]. Another U.S. investigation surveying 1853 veterans found 44% of post-9/11 veterans and 25% of pre-9/11 veterans experienced reintegration difficulties. Veterans who reported difficulties indicated they experienced strained family relationships, angry outbursts, and “feeling that they don’t care about anything” [11]. In another U.S. study, up to 72% of veterans reported at least one significant readjustment stressor such as marital problems or financial issues [12]. Canadian researchers identified 27% of Regular Force Veterans and 24% of Reserve Veterans of the Canadian Armed Forces reported a difficult adjustment to civilian life [13]. Additionally, a Danish study examining community reintegration among 454 Afghanistan veterans found that up to 18% experienced difficulties across multiple domains including social relationships, self-care, and general daily functioning [14]. Finally, a U.S. study found these reintegration difficulties appear to persist, with up to 62% of veterans reporting difficulties, despite data being collected an average of six years after separation - demonstrating the importance of understanding reintegration difficulties beyond the transition period [15].

Reintegration to civilian life following separation from full-time military service has also been recognised as a period of increased risk for the development of psychological disorders. A recent large-scale epidemiological study, jointly commissioned by the Australian Departments of Defence and Veterans’ Affairs, assessed the mental health and wellbeing of current (*n* = 8480) and ‘transitioned’ (*n* = 4326) Australian Defence Force (ADF) members who had separated from Defence within the last five years [16]. Findings revealed that transitioned service members had significantly poorer mental health compared to current serving ADF members across all domains measured, including psychological distress, posttraumatic stress disorder (PTSD), alcohol use, depressive symptoms, anxiety symptoms, suicidality, and anger. The researchers also found the prevalence of psychological distress among the transitioned ADF members was significantly higher than in the Australian community, (33.1% vs. 12.8% respectively). Further, a concerning 46% of transitioned service members met 12-month diagnostic criteria for a mental disorder using a structured diagnostic interview [16]. In addition to these findings, a large cohort study including 3.9 million Operation Enduring Freedom or Operation Iraqi Freedom deployed personnel investigated suicide risk among United States (US) service veterans. Results revealed an increased rate of suicide associated with separation from military service, while deployment itself was not associated with the rate of suicide [17]. Similarly, in their study of US veterans Pietrzak and colleagues [18] found that social support for veterans during the reintegration to civilian life was negatively associated with suicidal ideation. In line with this research, an Australian senate inquiry into rates of suicide in veterans highlighted the transition period as a critical time in the provision of intervention to reduce suicide risk [19].

The alarming findings above indicate that reintegration to civilian life is particularly challenging for service personnel, and can have a considerable impact on psychological health and wellbeing. A recent Productivity Commission inquiry into the ADF demonstrated that the median period of employment within the ADF is between seven to ten years for the three services, yet the “*stewardship of transition remains poor and supports have not improved in ways that are tangible to veterans*” (p.49). A key recommendation was to “*prepare members for other aspects of civilian life, including the social and psychological aspects of transition*” (p. 50) [20]. Despite this, evidence-based supports designed to specifically target psychological adjustment and reintegration post service are lacking. A central reason for the gap in evidence-based services is that no standardised psychometric measures have been developed to assist in the assessment of adjustment during the reintegration process. Without empirically developed measures, there is no applied and practical method to determine the factors that are associated with adjustment and successful reintegration in this population. This has consequently limited the ability to develop and assess targeted interventions to support reintegrating veterans.

While assessment of psychological adjustment is lacking, six questionnaires exist which measure other aspects of reintegration experiences and outcomes of military personnel. These include: (1) Post-Deployment Reintegration Scale [21]; (2) Combat-to-Home Transition Scale [4]; (3) Iraq Readjustment Inventory [22]; (4) Post-Deployment Readjustment Inventory [23]; (5) Community Reintegration of Service Members [24]; and (6) Military to Civilian Questionnaire [25]. All of these questionnaires were developed to evaluate the experiences of active duty military personnel returning from deployment [4, 21, 22, 25], or injured veterans specifically [24], not those permanently separating from the military – including both medical and voluntary separations. There may be unique experiences of military veterans permanently leaving service and returning to civilian society, yet much of the current literature groups post-deployment military personnel and ex-serving veterans together, and either assumes their reintegration experiences are similar or do not attempt to differentiate. The current measures cannot adequately assess this. Further, these questionnaires were developed and normed using U.S. and Canadian military populations and have not been validated with other nations or cultures. While some similarities may exist, the organisational and cultural aspects may vary between Defence Forces and as such, the above measures reflect factors that may not be applicable to a wider context.

To address this gap in both research literature and clinical and community practice, a mixed methods investigation of reintegration was undertaken. This study describes the development and validation of the self-report Military-Civilian Adjustment and Reintegration Measure (M-CARM), designed to assess psychological adjustment and cultural reintegration following permanent separation from the military, for all members, including voluntary and medical separations. The research was undertaken in two phases: Phase I – Item generation and refinement, and Phase II – Evaluation of factor structure and psychometric properties.

## Phase I – item generation and refinement

### Study objectives

Phase I of the study aimed to investigate the experience of psychological adjustment and cultural reintegration to civilian life following permanent separation from the military to facilitate item generation for the M-CARM. Specific objectives included:

1. Using interviews and focus groups, conduct a qualitative investigation of the lived experience of service veterans who have separated from the military.
2. Generate items for the M-CARM, based on thematic analysis of qualitative data and literature review.
3. Conduct an expert panel review of the measure as well as pilot testing with the target population to select refined items for further evaluation.

## Methods

### Participants

Participants were convenience samples of 60 ADF service veterans, 20 partners of ADF veterans, and 20 registered health care professionals working with veterans. Participants were excluded if they had current psychosis or if they were hospitalised for an unstable medical or psychological condition. Eligible veteran participants served in the regular ADF between 2006 and 2016 for a minimum of three years. Eligible partners were in a relationship with an eligible ADF veteran for a minimum of 12 months. Health care professionals were eligible if they had at least three years’ experience working with separated veterans. Table 1 provides an overview of participant demographic information.

**Table 1:** Demographic Characteristics of Phase I Sample

Demographic variables	Veterans (*n* = 60)	Partners (*n* = 20)	Health Professionals (*n* = 20)
Age, *M* (*SD*), range	43.72 (10.68), 25–67	41.21 (10.30), 23–61	
Gender, % (*n*)			
Female	13.3 (8)	95.0 (19)	25.0 (5)
Male	86.7 (52)	5.0 (1)	75.0 (15)
Service Type, % (n)			
Army	86.7 (52)		
Navy	3.3 (2)		
Air Force	10.0 (6)		
Rank, % (*n*)			
Commissioned Officers	33.3 (20)		
Non-Commissioned Officers	43.3 (26)		
Other Ranks	23.3 (14)		
Service duration, *M* (*SD*), range	17.53 (9.69), 3–40		
Deployed overseas, % (*n*)	73.3 (44)		
Medically discharged, % (*n*)	55.0 (33)		
Self-report PTSD, % (*n*)	51.7 (31)		
Received psychological treatment, % (*n*)	81.7 (49)		
Employment Status, % (*n*)			
Full-time	33.3 (20)		45.0 (9)
Part-time/Casual	8.3 (5)		50.0 (10)
Retired/pension	46.6 (28)		5.0 (1)
Unemployed	3.3 (2)		0.0 (0)
Other (e.g. student)	8.3 (5)		0.0 (0)
Missing	1.7 (1)		0.0 (0)
Years a health professional			3–27 years

### Procedure

Ethics approval for this study was obtained from the Department of Veterans’ Affairs Human Research Ethics Committee and Greenslopes Hospital Research and Ethics Committee. Participants were recruited via hospital and veteran community organisations. Staff members at these organisations were provided with study information for distribution among potential participants. Participants were also recruited through word of mouth and through social media, and publications and websites run by veteran community organisations. Those interested in participating were directed to contact the principal investigator. Potential participants were provided a study information sheet and interested participants were screened against eligibility criteria. If eligible, participants were then provided with a consent form and a mutually convenient time for an interview was scheduled. Written informed consent was obtained prior to interviews.

Veterans and partners completed one-on-one semi-structured interviews in person (*n* = 60) or over the phone (*n* = 20), while health professionals engaged in focus groups with the principal investigator at their place of work. Four focus groups were conducted with three to eight participants per group, and ranged from 36 to 77 min in length (*M* = 60.5). Focus group participants were asked a series of six open-ended questions exploring observations of veterans who have difficulty in civilian life, factors that prevent adjustment, and differences between military and civilian culture (see Additional file 1 for focus group questions). Interview questions were open-ended and designed to encourage understanding of the transition process and exploration of the reintegration experience, particularly in relation to common challenges faced when reintegrating from military culture to civilian culture as well as aspects that facilitate psychological adjustment (see Additional files 2 and 3 for interview questions). Length of interviews ranged from 52 to 229 min (*M* = 92.28). Interviews and focus groups were audio recorded and transcribed. Data were collected between March 2016 and December 2016.

Transcripts were reviewed by the principal investigator utilising thematic analysis which aims to identify, examine, and record patterns or “themes” within the interview transcripts [26]. The analysis process consisted of reading transcripts, identifying possible themes, and comparing and contrasting themes. This was performed through the process of coding in six phases to create established, meaningful patterns. These phases include familiarisation with data, generating initial codes, searching for themes among codes, reviewing themes, defining and naming themes, and producing a final report [26]. The data analysis program NVivo v.11,© QSR International, was used to assist in sorting and coding of the data. Analysis focused on identifying key trends relating to psychological adjustment and cultural reintegration and examining the differences between those who reintegrate to civilian life, with and without difficulty (ascertained throughout the course of the interviews), to determine factors that may indicate successful adjustment and reintegration.

Both inductive and deductive methods were used to generate items for the measure including a systematic literature review (see Romaniuk & Kidd, 2018 [27]) and the thematic analysis results obtained through the interviews and focus groups.

All items were reviewed for language, comprehensibility, and relevance as well as the face validity of the measure by an expert panel comprised of intended users including veteran health professionals (*n* = 9) and the target population of service veterans (*n* = 3). The veteran panel members were participants of the qualitative investigation who expressed an interest in providing ongoing feedback (demographic information is included in Table 1). Disciplines of the health professionals included clinical psychology (*n* = 3), social work (*n* = 1), occupational therapy (*n* = 2), mental health nursing (*n* = 2), and psychiatry (*n* = 1). Years of experience working with veterans as a health professional ranged from 10 to 27 years. Following this review, an additional convenience sample of service veterans (*n* = 11) piloted both the remaining 49 items as well as the online platform used in Phase II. These participants were invited to provide open-ended comments on the measure as well as any other aspect of the online survey.

## Results

Qualitative data was analysed to extract key themes [26] related to adjustment and reintegration with particular attention on common factors among those veterans who had described adjusting well to life after service versus those who had not. This analysis, along with a systematic literature review [27], informed the generation of 53 candidate items. Items reflected themes of social connectedness and belonging, attitudes towards civilians and military service, attitudes towards help seeking, sense of identity and purpose, as well as rigidity and psychological flexibility. The findings of the literature review established a central theme of ‘loss’ in three key areas: culture and community, identity and purpose. The qualitative study echoed these findings as themes reiterated that military culture and differences with civilians, identity, purpose as well as belonging were central to psychological adjustment following military service. Items 43, 30, 46, 38, 47, 14, 26, 15, 36, and 17 were derived from these themes. A number of novel themes (that weren’t apparent in the systematic literature review) were also found in the qualitative analysis, which appeared to strongly contribute to successful reintegration to civilian life. These included; help seeking attitudes and behaviours, resentment and regrets about experiences during service, psychological flexibility and ability to adapt. Items derived from these themes included: 34, 21, 1, 40, 12, 20, 41, 42, 32, 5, and 3. The expert panel provided open-ended written feedback to the first author via e-mail. This led to the removal of four items due to relevance and/or redundancy and re-wording of one item for clarity. Following this, the remaining 49 items were administered to a small sample of veterans (*n* = 11) and another two items removed. The Likert scale response options were acceptable to all respondents/reviewers. The remaining 47-item M-CARM was subsequently evaluated in Phase II.

## Phase II – evaluation of factor structure and psychometric properties

### Study objectives – phase II

Phase II of the study evaluated the refined item set of the developed measure in a sample of Australian veterans to determine the factor structure and evaluate the psychometric properties of the scale. Specific objectives included:

1. Determine the factor structure of the M-CARM via Exploratory Factor Analysis (EFA), in a random split-half sample.
2. Test the hypothesized EFA factor structure of the M-CARM via Confirmatory Factor Analysis (CFA), in the other half of the sample.
3. Evaluate reliability of the refined M-CARM item set using indices of internal consistency and temporal stability.
4. Evaluate evidence for the validity of the M-CARM through convergent, divergent and discriminant validity.

## Methods

### Participants

Participants were 725 veterans over the age of 18 who had permanently separated from the ADF during or after the year 2000. See Table 2 for an overview of participant demographic information. The sample was predominately male (78.5%), served in the Army (57.8%), and from Non-commissioned/Other ranks (73.7%). While this was a convenience sample, these statistics are comparable to the demographic breakdown of the total ADF in terms of gender, rank and service branch (See Table 3) [28].

**Table 2:** Demographic Characteristics of Phase II Sample

Demographic variables	Full sample (*n* = 725)
Age, *M* (*SD*), range	44.57 (9.94), 21–70
Gender, % (*n*)	
Female	21.4 (155)
Male	78.5 (569)
Did not disclose	0.1 (1)
Marital status	
Single	20.6 (149)
Married	60.7 (440)
Partner/De facto	18.8 (136)
Highest level of education	
No education	0.3 (2)
Secondary	53.5 (388)
University	46.2 (335)
Service Type, % (*n*)	
Army	57.8 (419)
Navy	20.0 (145)
Air Force	18.8 (136)
More than one	3.4 (25)
Rank, % (*n*)	
Other Ranks	73.7 (534)
E02	13.0 (94)
E03	6.6 (48)
E04	2.8 (20)
E05	20.8 (151)
E06	14.1 (102)
E07	–
E08	11.2 (81)
E09	4.8 (35)
E10 Officer Cadet	0.4 (3) -
Officer	21.7 (157)
O-1	0.1 (1)
O-2	0.7 (5)
O-3	3.4 (25)
O-4	8.6 (62)
O-5	2.9 (21)
O-6	5.7 (41)
O-7	0.1 (1)
O-8	0.1 (1)
O-9	–
O-10	–
Unknown	4.7 (34)
Years of service, *M* (*SD*) range	15.64 (9.16), 1–49
Years since separation	8.04 (6.67), 0–19
Deployed to combat zone, % (*n*)	64.4 (467)
Medically discharged, % (*n*)	35.4 (257)
Self-report psychological condition, % (*n*)	
Yes	57.5 (417)
No	35.4 (257)
Unsure	7.0 (51)
Provisional PTSD diagnosis, % (*n*)	40.4 (293)
Received psychological treatment, % (*n*)	
Yes	58.8 (426)
No	4.7 (34)
Unsure	1.1 (8)
Not applicable	35.4 (257)
Employment Status, % (*n*)	
Full-time	53.2 (386)
Part-time/Casual	11.6 (84)
Retired/pension	23.0 (167)
Unemployed	8.3 (60)
Other (e.g. student, volunteer)	3.9 (28)
Submitted Veteran Affairs claim, % (*n*)	68.3 (495)

*Note.* Provisional PTSD diagnosis made in line with PTSD Checklist for DSM-5 criteria

**Table 3:** Phase II Sample characteristics in comparison to ADF statistics

Demographic Variables	Permanent ADF personnel* (*n* = 58,380)	Phase II Sample (*n* = 725)
Age (*M, SD*, Range)	31 (−)	44.57 (9.94), 21–70
Gender		
Male	81.4 (47,263)	78.5 (569)
Female	18.6 (10,760)	21.4 (155)
Did not disclose	0.1 (5)	0.1 (1)
Service Type, % (*n*)		
Army	51.36 (29,982)	57.8 (419)
Navy	24.28 (14,176)	20.0 (145)
Air Force	24.36 (14,222)	18.8 (136)
More than one	–	3.4 (25)
Rank, % (*n*)		
Other Ranks	73.8 (42,863)	73.7 (534)
Officers	26.2 (15,196)	21.7 (157)
Unknown	–	4.7 (34)

*Note.* *Data for Permanent ADF personnel (*n* = 58,380) was obtained from the Defence Annual Report 2018–19 [28]

Participants were excluded if they were currently serving in the reserves, or had an unstable medical or psychological condition (indicated by current hospitalisation). Participants were recruited for the study through notices to ex-service organisations as well as hospital and community service providers throughout Australia. Posts and paid advertisements for the study were circulated on the Gallipoli Medical Research Foundation’s (GMRF) social media pages (Facebook and LinkedIn). Study information was also circulated to veterans who had elected to be contacted by GMRF about future research studies.

### Procedure

Ethics approval for this study was obtained from the Department of Defence and Veterans’ Affairs Human Research Ethics Committee. Questionnaires were completed on the online survey platform Web Survey Creator. No incentives were offered for participating. On accessing the survey, participants completed screening questions pertaining to eligibility criteria. Eligible participants were presented with a detailed information sheet about the study and indicated their written consent before proceeding to questionnaires administered to participants in the order shown in Measures. A subset of participants were invited to complete the M-CARM a second time between one to two weeks after completing the initial survey to assess temporal stability (response rate = 86.27%). Data were collected between March 2018 and September 2019.

### Measures

#### Demographic questionnaire

Participants completed questions pertaining to demographic characteristics, their psychological and physical health, and their service history.

#### Reintegration questions

Participants were asked the following questions related to their reintegration: (a) Have you found it easy to find and retain employment since leaving the military? (Y/N or NA due to retirement age or medically retired), (b) Did you find your transition out of the military difficult? (Y/N), and (c) Do you think you have reintegrated or adjusted back to civilian life? (Y/N).

#### Military-civilian adjustment and reintegration measure (M-CARM)

The preliminary M-CARM consisted of 47 items developed during Phase I of this study. Items reflect themes of social connectedness and belonging, attitudes towards civilians and military service, attitudes towards help seeking, sense of identity and purpose, and psychological flexibility. To administer the M-CARM, responders are asked the following: *Please read each statement carefully and indicate how much you agree or disagree. Please consider your responses honestly; there are no right or wrong answers. If you find some of the questions difficult, please give the answer that is true for you most of the time.* Followed by questions rated along a 5-point Likert scale (1 = *Disagree*, 2 = *Slightly Disagree*, 3 = *Neither Agree or Disagree*, 4 = *Slightly Agree*, 5 = *Agree*).

#### PTSD checklist for DSM-5 (PCL-5)

The PCL-5 [29] is a 20-item self-report questionnaire that assesses the 20 DSM-5 symptoms of PTSD and provides a total score reflecting severity. The PCL-5 has demonstrated strong psychometric properties and is used regularly for research purposes including screening individuals for PTSD and making a provisional PTSD diagnosis [30–32].

#### Depression anxiety stress Scale-21 (DASS-21)

The DASS-21 [33] is a 21-item self-report questionnaire that assesses the severity of depressive, anxiety, and stress symptoms and provides scores for each. The sub-scales have been well validated within previous research and has strong psychometric properties [33–35].

#### The World Health Organization quality of life instrument (WHOQOL-BREF)

The WHOQOL-BREF [36] is comprised of 26 items that assess quality of life across four domains: physical health, psychological health, social relationships, and environment. In addition to the four subscale scores, it also contains a global quality of life item, in which responders rate their overall quality of life on a 5-point Likert scale (very poor to very good). This cross-cultural instrument has been normed for a wide range of populations and has demonstrated excellent psychometric properties [36–38].

#### Walter reed functional impairment scale (WRFIS)

The WRFIS [39] consists of 14 items designed to assess overall functional impairment and includes scores for domains that are not used in this analysis: social, physical, occupational, and personal functioning. This scale was designed for use in military populations and has previously demonstrated excellent psychometric properties [39].

#### Military to civilian questionnaire (M2C-Q)

The M2C-Q [25] is a 16-item self-report questionnaire of post-deployment community reintegration difficulties (vs. focusing on difficulties after permanent separation from the military). It provides a total score that covers a range of reintegration domains including interpersonal relationships, productivity at work, in school or at home, community participation, self-care, leisure, and perceived meaning in life. The questionnaire has demonstrated good psychometric properties in previous research [2, 25].

#### Nightmare distress questionnaire (NDQ)

The NDQ [40] is a 13-item self-report questionnaire which yields a total score that evaluates the emotional disturbance attributed to nightmares as well as nightmare-related symptoms. The questionnaire has demonstrated good psychometric properties in previous research [41].

### Statistical analysis

Analyses were conducted using the IBM SPSS version 24, IBM AMOS version 21, and R [42]. Frequencies, descriptive statistics (e.g., means, standard deviations, skewness and kurtosis), and graphs of each variable were produced. Data were screened for data entry errors, outliers, and violations of assumptions. No missing data was found within measures (due to the set up of the online questionnaire preventing incomplete responses) and no problematic outliers were found. Participants were able to close survey at any time and end participation prior to completion of all measures. As a result, 90.5% completed the entire survey, while the remainder only completed the M-CARM as well as demographic and reintegration questions. Following data screening and cleaning, frequencies and descriptive statistics of all demographic and military service variables were produced. Reverse-scored items were re-coded prior to analysis.

#### Factor analyses

An EFA with oblique rotation was conducted to explore underlying latent constructs and factor structure of the M-CARM. To verify sampling adequacy for the analysis, inspection of the Kaiser-Meyer-Olkin (requires a value > .5) and all elements on the diagonal anti-image matrix of covariances and correlations (requires a value > .5) [43] were conducted. Bartlett’s test of sphericity was inspected to ensure intercorrelation between variables (requires *p* < .05) and multicollinearity detected via the determinant of the correlation matrix (requires a value > 0.00001). Factor extraction was determined by inspecting Cattel’s scree plot and retaining only factors with an eigenvalue > 1 (Kaiser’s criterion). The number of factors retained was determined by (a) inspecting the variance explained by each factor; (b) exploring the loadings of items on each factor by examining for values > .30, cross-loading and non-loading items; (c) identifying factors with only one or two items; and (d) underlying theory and intended use of the measure.

A CFA through structural equation modeling using maximum likelihood estimation was then conducted on a randomised split half sample to test the hypothesized factor structure found in the EFA. Chi-Square (*χ*^*2*^), Root Mean-Square Error of Approximation (RMSEA), Comparative Fit Index (CFI), Tucker-Lewis Fit Index (TLI) and Normed Fit Index (NFI) were the indices used to assess the fit of the hypothesized model to the data. Such fit-indices were used in addition to the traditional *χ*^*2*^ test, as *χ*^*2*^ has been shown to be highly sensitive to slight deviations from perfect fit in large samples [44]. CFI and TLI of .95 and above [45], RMSEA of .05 and below [46] and a NFI of .90 and above indicate excellent fit to the data [47, 48]. Acceptable model fit was determined using cut-off values of ≥ .90 for CFI and TLI and values ⩽ .08 for RMSEA [48].

#### Reliability

Reliability was evaluated via internal consistency and temporal stability. Internal consistency was assessed using Cronbach’s alpha with the following interpretation: .90 or above = excellent, .89 to .80 = good, .79 to .70 = acceptable, .69 to .60 = questionable, .59 to .50 = poor, and below .50 = unacceptable [49]. Temporal stability for the total and factor scores was assessed using test-retest reliability coefficient on the subset of participants (*n* = 186) who completed the M-CARM at two time points between seven and 14 days apart. These scores were correlated to produce a test-retest reliability coefficient (Pearson’s correlation coefficient) with higher coefficients indicating greater reliability.

#### Validity

In addition to the factor analyses, construct validity was evaluated through convergent, divergent and discriminant validity. Pearson’s correlation coefficients between the total M-CARM scores and total or subscale scores from other validated questionnaires related to mental health and functioning were calculated to assess the direction and strength of these relationships. High coefficients with related constructs (convergent validity) and lower coefficients with unrelated constructs (divergent validity) represent stronger evidence of construct validity. It was predicted that the M-CARM total score would strongly and negatively correlate with the M2C-Q – a measure of functioning post deployment developed specifically for the military population. It was also predicted that the M-CARM would moderately to strongly negatively correlate with the DASS-21and WRFIS and positively with the WHO-QOL, as based on previous research it is empirically and clinically sound to expect that psychological adjustment to life after military service would be related to broad experiences of mental health and general functioning [16, 50]. In comparison, it was predicted that the M-CARM would correlate negatively and at lower magnitude with the NDQ compared to other measures, given the specific focus of this measure on sleep.

Discriminant validity refers to the tool’s ability to distinguish between groups that it should theoretically or clinically be able to distinguish between. To assess this, *t*-tests for mean differences on the M-CARM total score were conducted on five binary variables reflecting overall post-separation adjustment: the three reintegration questions (yes/no), provisional diagnosis of PTSD based on the PCL-5 (yes/no) and the WHO-QOL global quality of life question (“poor/very poor” vs. “good/very good”).

## Results

### Factor analyses

To assess the factor structure of the M-CARM, the sample was randomly split into two groups to enable an EFA (*n* = 357), and a CFA (*n* = 368). No significant differences were present across demographic variables and clinical severity scores between the EFA and CFA samples. Reverse-scored items were re-coded prior to analysis.

#### Exploratory factor analysis

An EFA with Principal Axis Factoring and oblique oblimin rotation was conducted to explore underlying latent constructs of the M-CARM. Sampling adequacy was verified by a Kaiser-Meyer-Olkin value of .955. In addition, all elements on the diagonal anti-image matrix of covariances and correlations demonstrated values greater than .5, except for item 11(.499), which was subsequently removed. Bartlett’s test of sphericity demonstrated adequate intercorrelations between variables, *χ*^*2*^_1081_ = 9736.83, *p* < .001. Eight factors were extracted with eigenvalues greater than one (1.11–17.52), explaining 53.72% of the variance. The point of inflection on Cattel’s scree plot suggested 3–4 factors. Inspection of the pattern matrix demonstrated a number of non-loading (loadings < .30), and relatively low-performing items (loadings < .40). A series of EFAs were run removing these items until all factors consisted of items with loadings greater than .40, resulting in the removal of 22 items. One item cross-loaded onto factors 1 and 3, and an EFA was run with this item removed. Factors composed of more than four items were then examined for potential item removal in order to refine and simplify the scale. Guided by conceptual reasoning and consideration of factor loadings, a further three items were removed, and the EFA was rerun. The 21-item M-CARM resulted in a 5-factor solution that explained 53.22% of the variance, demonstrating greater parsimony with considerably fewer items and factors than the initial solution. See Table 4 for item-total correlations, communalities, and factor loadings for the 21-item M-CARM. To determine if group differences were present or variation in factor structure for those more recently separated from the ADF, additional exploratory factor analyses were run on those who had been separated from Defence up to 3 years (i.e. during or after the year 2016; *n* = 207) as well as up to 5 years (i.e. during or after the year 2014; *n* = 301). The five-factor structure was replicated in both samples, with all items loading on the equivalent factors as the overall sample (see Additional files 4 and 5 for factor loadings and sub-sample demographic information).

**Table 4:** Item–Total Correlations, Extracted Communalities and Oblique Rotated Five-Factor Solution for the 21-Item M-CARM (*n* = 357)

Item	Item-total correlation	*h*^2^	Factors				
			1	2	3	4	5
43. I have things that give me a sense of purpose, outside of paid employment.	0.60	0.66	0.85^a^	0.03	−0.06	−0.04	−0.02
30. I have interests and hobbies that are enjoyable or meaningful.	0.58	0.60	0.80^a^	−0.02	−0.07	0.03	−0.01
46. I have a sense of purpose.	0.71	0.70	0.76^a^	0.02	0.11	0.08	−0.04
38. I am fulfilled.	0.74	0.68	0.65^a^	0.01	0.15	0.08	0.09
47. I feel I don’t belong anywhere.	0.72	0.63	0.60^a^	0.05	0.16	0.08	0.09
14. Outside of the military, I have found people that I connect with through shared interests or beliefs.	0.60	0.45	0.54^a^	0.07	0.08	−0.02	0.14
34. I would ask for help if I needed it.	0.46	0.73	0.16	0.82^a^	−0.07	− 0.07	0.08
21. I would never seek help from a mental health professional.	0.21	0.49	−0.19	0.73^a^	0.01	0.01	0.01
1. I know how to access professional support for my health.	0.30	0.29	0.09	0.49^a^	0.06	0.04	−0.12
40. I find it difficult to ask for help if I’m struggling.	0.48	0.39	0.12	0.48^a^	0.03	0.04	0.16
26. Despite all my experience in the military, I am undervalued by civilians.	0.60	0.64	0.10	−0.001	0.75^a^	0.03	−0.03
15. Civilians are disrespectful and rude.	0.58	0.55	0.01	0.03	0.65^a^	0.04	0.11
36. Civilians seem to be concerned with trivial matters.	0.44	0.43	− 0.01	− 0.004	0.63^a^	−0.13	0.15
17. I don’t think society puts much value on military service and experience.	0.46	0.42	0.02	0.05	0.60^a^	0.16	−0.14
12. I’m angry about the way I was treated during my service.	0.43	0.71	−0.08	0.03	−0.04	0.89^a^	−0.03
20. The military broke me and then kicked me out.	0.55	0.55	0.12	−0.06	−0.002	0.63^a^	0.15
41. I have a lot of regrets about my service.	0.44	0.30	0.08	0.05	0.08	0.43^a^	0.04
42. I am more regimented than flexible.	0.52	0.60	−0.01	0.06	− 0.01	0.05	0.76^a^
32. I find it difficult to change once I have a set routine.	0.52	0.54	0.02	−0.01	0.10	0.04	0.66^a^
5. I am a flexible person and I don’t mind changing to suit others when required.	0.52	0.41	0.27	0.01	−0.01	0.05	0.45^a^
3. Some of my military habits cause problems for me.	0.54	0.40	0.02	0.01	0.21	0.15	0.42^a^
Variance explained (%)			33.05	6.57	5.78	4.35	3.47
Eigenvalues			7.38	1.86	1.65	1.35	1.19

*Note.* ^a^Loadings > 0.40

#### Confirmatory factor analysis

Structural equation modeling via CFA was used to test the hypothesized 21-item, 5-factor structure found in the EFA. The results demonstrated that both the 5-factor model, with correlated factors, and the 5-factor model with a higher order factor, obtained acceptable to excellent fit indices without modification; *χ*^*2*^ = 299.51 *df* = 155; CFI = .96, TLI = .94, NFI = .91, RMSEA = .05. and *χ*^*2*^ = 304.96, *df* = 160; CFI = .96, TLI = .94, NFI = .91, RMSEA = .05, respectively.

All items loaded on the specified factors in line with EFA results (see Table 5). Factor 1, *Purpose and Connection* is comprised of six items that assess veterans’ sense of purpose, fulfillment, belonging and achievement through participation in meaningful activities and establishing social connections outside of the military. Factor 2, *Help seeking* is comprised of four items and assesses veterans’ attitude towards seeking help for health and mental health difficulties, as well as knowledge about accessing professional support and propensity to ask for help if needed. Factor 3, *Beliefs about civilians* is comprised of four items and assesses veterans’ beliefs and perceptions about the values and behaviours of civilians and general society, including attitudes towards, and treatment of, military personnel. Factor 4, *Resentment and Regret* is comprised of three items that assesses veterans’ animosity toward the military and their experience of service and separation. Finally, Factor 5, *Regimentation* is comprised of four items and assesses veterans’ flexibility and adaptability to change, particularly with regard to routines or in response to others, as well as retention of problematic military habits. It contains 13 reverse coded items (noted Table 5).

**Table 5:** Factor Loadings for the Five-Factor CFA Model, with Higher Order Factor

Item	Factor loadings				
	1	2	3	4	5
43. I have things that give me a sense of purpose, outside of paid employment.	.70				
30. I have interests and hobbies that are enjoyable or meaningful.	.67				
46. I have a sense of purpose.	.84				
38. I am fulfilled.	.81				
47. I feel I don’t belong anywhere. (R)	.85				
14. Outside of the military, I have found people that I connect with through shared interests or beliefs.	.61				
34. I would ask for help if I needed it.		.81			
21. I would never seek help from a mental health professional. (R)		.57			
40. I find it difficult to ask for help if I’m struggling. (R)		.48			
1. I know how to access professional support for my health.		.61			
36. Civilians seem to be concerned with trivial matters. (R)			.57		
26. Despite all my experience in the military, I am undervalued by civilians. (R)			.93		
15. Civilians are disrespectful and rude. (R)			.77		
17. I don’t think society puts much value on military service and experience. (R)			.56		
12. I’m angry about the way I was treated during my service. (R)				.77	
20. The military broke me and then kicked me out. (R)				.92	
41. I have a lot of regrets about my service. (R)				.76	
42. I am more regimented than flexible. (R)					.59
32. I find it difficult to change once I have a set routine. (R)					.57
5. I am a flexible person and I don’t mind changing to suit others when required.					.56
3. Some of my military habits cause problems for me. (R)					.84

*Note*. Factor 1 = Purpose and Connection; Factor 2 = Help seeking; Factor 3 = Beliefs about civilians; Factor 4 = Resentment and Regret; Factor 5 = Regimentation. Reverse-scored items are denoted with an (R)

**Fig. 1:**
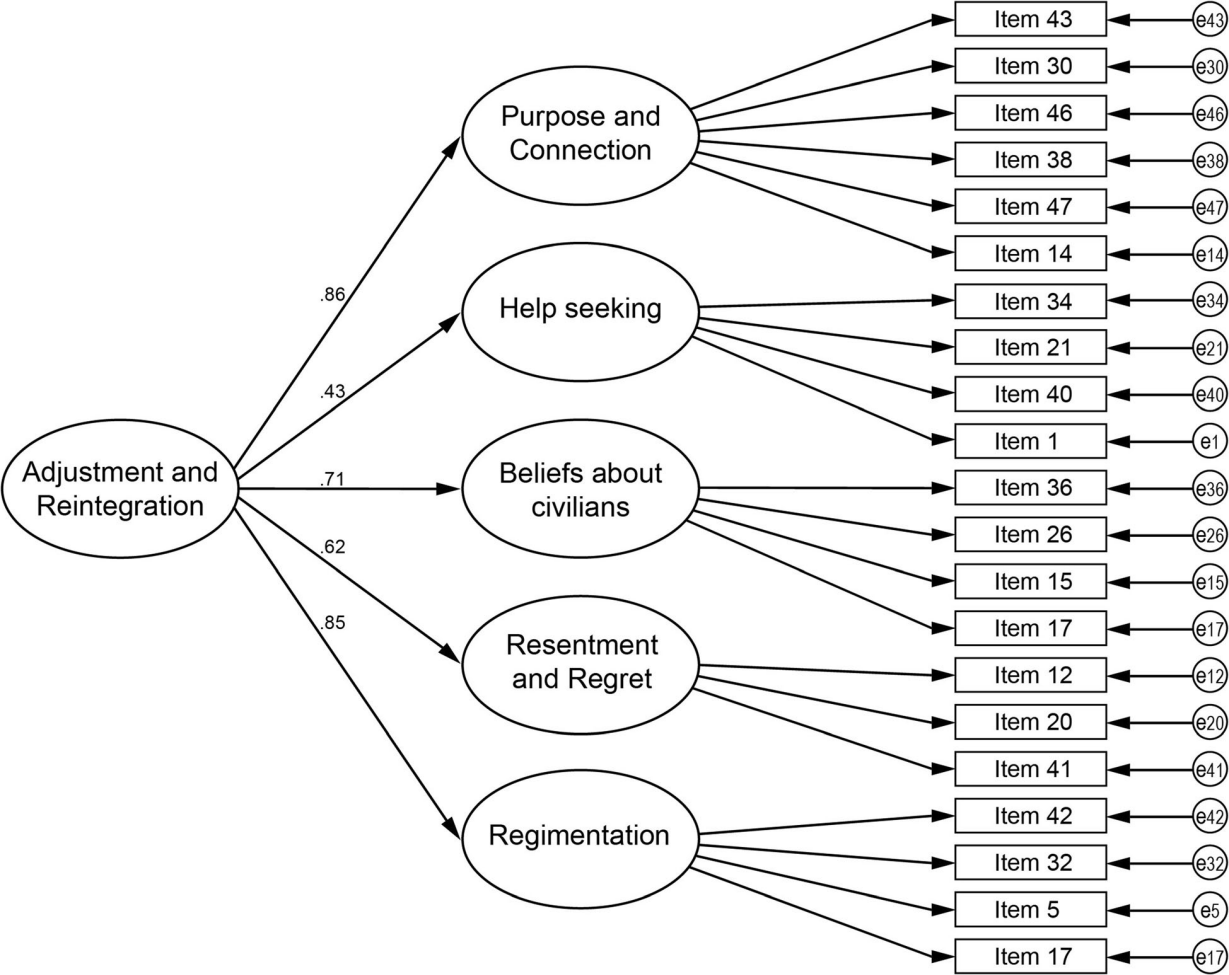
Five-Factor model with a Higher Order Factor of Adjustment and Reintegration

Given there was no significant difference between models (*p* = .36), and no differences between fit indices, the higher order model was considered the preferred model as it conceptually and theoretically demonstrates a stronger representation of the underlying constructs with the inclusion of the global factor of *Adjustment and Reintegration*. Schematic representation of this model, including loadings of each factor onto the higher order factor, is presented in Fig. 1. The *M*, *SD* and range of each Factor as well as total M-CARM scores appear in Table 6.

**Table 6:** M, SD, range and internal consistency for M-CARM factors and total score

Factor	Descriptives			Temporal Stability
	*M*	*SD*	Range	Test-retest Correlation
1. Purpose and connection	19.71	6.95	6–30	.91
2. Help seeking	15.18	3.79	4–20	.77
3. Beliefs about civilians	9.21	4.08	4–20	.89
4. Resentment and regret	9.13	3.95	3–15	.85
5. Regimentation	10.90	4.08	4–20	.84
Total M-CARM	64.13	16.84	26–105	.93

#### Reliability evaluation

Internal consistency was investigated with the full sample of M-CARM completers (*n* = 725) and Cronbach’s alpha of .90 was found for the total 21-item M-CARM, with a range of .74 to .90 for each of the five factors. A subset of 186 participants completed the M-CARM twice, between seven and 14 days apart. The 21-item M-CARM total scores were strongly correlated between Time 1 and Time 2, *r* = .93 (*p* < .001). Each of the five M-CARM factors were also significantly correlated between time points (all *p* < .001; see Table 6).

#### Validity evaluation

A subset of 656 participants (90.5% of the total sample) completed demographic measures, the M-CARM, and the additional measures to enable validity analysis. This subset of complete cases did not significantly differ on any demographic variables, clinical scores, validation measures, M-CARM total score or subscale scores compared incomplete cases (i.e. those who only completed the demographic and M-CARM questions).

##### Convergent and divergent validity

Correlations between total scores on the M-CARM and the WRFIS, M2C-Q and NDQ as well as subscale scores on the DASS-21 and WHO-QOL are presented in Table 7. All correlations were found to be significant (*p* < .001), and in the expected direction. Results also demonstrated the M-CARM total score was most strongly correlated with the M2C-Q (*r* = −.80) and the Psychological subscale of the WHO-QOL (*r* = .76), and most weakly correlated with the NDQ (*r* = −.55) relative to the other measures. Strong correlations were found between the M-CARM and the DASS-21 Depression subscale (*r* = −.71), and the WRFIS (*r* = −.70) and moderate correlations with the Physical (*r* = .66), Social relationships (*r* = .62) and Environmental (*r* = .68) subscales of the WHO-QOL as well as the Stress (*r* = −.66) and Anxiety (*r* = −.59) subscales of the DASS-21.

**Table 7:** Correlation Between M-CARM Scores and Validated Psychometric Measures (*n* = 656)

Measure	1	2	3	4	5	6	7	8	9	10	11
1. M-CARM Total	–										
2. DASS-21 Depression	−0.71	–									
3. DASS-21 Anxiety	−0.59	0.72	–								
4. DASS-21 Stress	−0.66	0.79	0.81	–							
5. WRFIS Total	−0.70	0.78	0.73	0.74	–						
6. WHO-QOL Physical	0.66	−0.70	− 0.64	− 0.66	− 0.83	–					
7. WHO-QOL Psychological	0.76	−0.83	− 0.62	− 0.70	− 0.77	0.75	–				
8. WHO-QOL Social	0.62	−0.62	− 0.43	− 0.51	− 0.59	0.59	0.70	–			
9. WHO-QOL Environmental	0.68	−0.61	− 0.52	− 0.54	− 0.63	0.65	0.69	0.60	–		
10. M2C-Q Total	−0.80	0.83	0.71	0.78	−0.89	−0.78	− 0.83	−0.67	− 0.68	–	
11. NDQ Total	−0.55	0.64	0.76	0.69	−0.70	−0.64	− 0.57	−0.44	− 0.47	0.68	–

*Note. M-CARM* Military-Civilian Adjustment and Reintegration Measure, *DASS-21* Depression, Anxiety and Stress Scale-21, *WRFIS* Walter Reed Functional Impairment Scale, *WHO QOL* =World Health Organization Quality of Life Scale Brief, *NDQ* Nightmare Distress Questionnaire. All *p*’s < .001

##### Discriminant validity

Mean M-CARM scores and *t*-test results for binary reintegration variables and PTSD diagnosis are presented in Table 8. For all variables there was a significant difference of large magnitude (*d* > 1.0) found in M-CARM scores between groups (all *p* < .001), with those reporting difficulties finding employment, difficulties with the transition experience and not adjusting to life post-service exhibiting significantly lower mean scores on the M-CARM compared to those reporting no difficulties. Further, those that obtained a provisional diagnosis of PTSD on the PCL-5, also exhibited significantly lower scores on the M-CARM. Finally, those who reported poor/very poor quality of life exhibited significantly lower M-CARM scores compared to those who reported good/very good quality of life (*p* = <.001; *d* > 1.0) see Table 9).

**Table 8:** Mean M-CARM Scores and t-test Results for Binary Variables

Reintegration questions and PCL-5	Yes			No			*t*-test	Effect Size
	*n*	*M*	*SD*	*n*	*M*	*SD*	*p*	*Cohen’s d*
Question 1^a^	274	73.13	15.74	294	57.08	14.09	<.001	1.07
Question 2	513	60.10	15.03	143	79.21	14.52	<.001	1.29
Question 3	328	74.17	14.71	328	54.36	12.53	<.001	1.45
Provisional PTSD diagnosis	280	53.44	12.57	376	72.32	15.07	<.001	1.36

*Note.* Question 1: Have you found it easy to find and retain employment since leaving the military? ^a^not applicable to some participants (i.e. compulsory retirement age or medically discharged; *n* = 568). Question 2: Did you find your transition out of the military difficult? Question 3: Do you think you have reintegrated or adjusted back to civilian life?

**Table 9:** Mean M-CARM Scores and t-test Results for WHO-QOL Global Item

WHO-QOL item	Poor/Very poor (1–2)			Good/Very good (4–5)^a^			*t*-test	*Effect size*
	*n*	*M*	*SD*	*n*	*M*	*SD*	*p*	*Cohen’s d*
Overall quality of life	161	49.40	11.67	326	74.67	14.31	<.001	1.94

*Note.* ^a^Analysis excluded neutral responses (3) to the WHO-QOL global item

## Discussion

The M-CARM was developed to address the growing need to understand and identify service veterans who have difficulty reintegrating from military service to civilian life, and the lack of psychometric instruments that assess psychological adjustment and cultural reintegration to society following permanent separation from the military. This measure underwent a rigorous development process including a qualitative study investigating reintegration experiences and identification of potential factors associated with successful reintegration, as well as a systematic literature review of psychological adjustment post-separation.

### Factor structure

Following both inductive and deductive methods to generate items for the M-CARM, an EFA was conducted resulting in a 21-item, 5-factor solution comprised of the following: Purpose and Connection, Help seeking, Beliefs about civilians, Resentment and Regret, and Regimentation. This hypothesized factor structure, with a higher order factor of Adjustment and Reintegration, was tested and verified by CFA in a separate sample demonstrating an acceptable to excellent fit to the data. This factor structure, and verification of underlying constructs, enables the calculation of both subscale scores (factors 1–5) and a total score, which enhances clinical utility.

### Reliability and validity

Reliability evaluation of the M-CARM demonstrated excellent internal consistency through a high Cronbach’s alpha for the total M-CARM score as well as acceptable to excellent internal consistency for each subscale. Excellent temporal stability via high test-retest correlations for total M-CARM scores as well as each of the five subscales between time points was also found.

The M-CARM also demonstrated good convergent and divergent validity through examination of the relationship with other psychometric measures. As predicted, the M-CARM was most strongly related to the M2C-Q. This is in line with the theoretical underpinnings of the measures as both the M-CARM and M2C-Q have been developed for the target population of military veterans in relation to a period of reintegration, while the M-CARM also addresses psychological adjustment and cultural aspects in relation to permanent separation. Of note, the M2C-Q demonstrated a stronger relationship to the military developed measure of daily functioning (WRFIS) compared to the relationship between the M-CARM and the WRFIS. Given the single factor M2C-Q has a greater emphasis on general functioning, while the M-CARM has a multifaceted approach to reintegration, the observed correlations align with underlying constructs of each scale and demonstrate unique contributions of the two military-reintegration themed measures.

Further, as predicted, the M-CARM demonstrated a relatively weaker relationship with the NDQ, compared to the other measures. This was also expected due to the specific nature of the NDQ in assessing the experience of nightmares, rather than psychological adjustment or the experience of reintegration for veterans. It was also predicted that the M-CARM would moderately to strongly correlate with the DASS-21, WHO-QOL, and WRFIS based on previous research linking the period of transition for veterans and experiences of mental health and general functioning [16, 50]. Results were consistent with this prediction, with significant moderate to strong correlations establishing that participants with higher scores on the M-CARM (indicating better adjustment and reintegration) reported better quality of life and participants with lower scores on the M-CARM (indicating poorer adjustment and reintegration), reported higher scores on depression, anxiety, stress, nightmares and difficulties with daily functioning.

Finally, discriminant validity was indicated with the M-CARM demonstrating significant distinction between groups on reintegration, mental health, and quality of life variables. Participants’ scores on the M-CARM were significantly lower if they reported difficulties with finding employment post-service, difficulties during the transition process, and if they reported they had not adjusted to life after military service. Participants with a provisional diagnosis of PTSD also had significantly lower M-CARM scores, as well as those who rated their quality of life as poor or very poor.

### Initial scoring guidelines

**Fig. 2:**
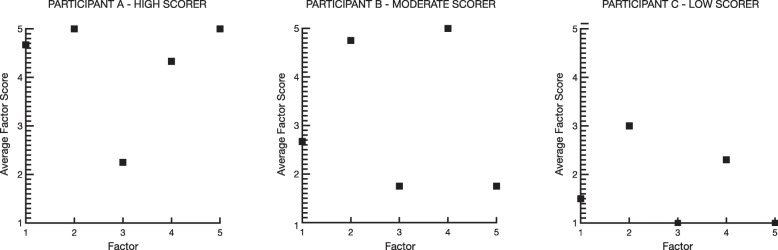
M-CARM Scoring Profile Examples

To score the M-CARM, 13 items are reversed-scored and a total adjustment and reintegration score is derived from summing the 21-items. Scores range from 21 to 105, with higher scores indicating better psychological adjustment and cultural reintegration. To enable detection of key areas of need, a scoring profile can be created by calculating an average score for each subscale and plotting values in relation to other subscale scores. See Fig. 2 for examples of scoring profiles from the current sample, including three randomly selected participants with high, moderate and low total M-CARM scores. Low subscale scores indicate areas of need and may be used to assist treatment planning and sequencing of reintegration support. Participant A obtained a high total score on the M-CARM, and subscale results indicate only one potential area of need: beliefs about civilians. However, this individual is likely to be adjusting well to civilian life overall, so may not necessitate intervention. Participant B obtained a moderate total M-CARM score, and their results indicate that they may benefit from support in three areas: finding purpose again and building social connections, adjusting their potentially unhelpful beliefs about civilians and society, as well as addressing regimentation by improving psychological and behavioural flexibility. Finally, Participant C obtained a low total score on the M-CARM and subscale results indicate they may benefit from support in all five of the areas of adjustment and reintegration. Scores indicate that beliefs about civilians and regimentation are particularly challenging for this individual, and are indicative of areas to prioritize during intervention planning.

### Implications of results

Overall, the results provide initial evidence that the M-CARM is a valid and reliable tool to aid the assessment of psychological adjustment and cultural reintegration after permanent separation from military service. Given the overwhelming evidence indicating that the period of transition out of service is particularly challenging for a substantial proportion of service veterans, which persists for years after separation [16], this tool adds a vital method of evidence-based individualized assessment indicating progress towards successful reintegration (irrespective of time since separation). This measure has both promising research and clinical utility within the veteran population, as there are currently no other tools to assess adjustment and reintegration following permanent separation from the military. All other transition measures currently available focus on community reintegration following deployment and/or exclusively injured veterans and not permanent separation of all service personnel.

This measure has the potential to contribute to further research regarding the relationship between mental health conditions and reintegration post-service, as well as potentially determining how current mental health treatments may address the key factors that appear fundamental for an adaptive and successful reintegration to civilian life. The M-CARM may also be useful in clinical and community settings including government and non-government veteran-based counselling services and reintegration support services as both a tool to indicate key areas of potential need for intervention planning to improve adjustment post service, as well as a method of determining reintegration progress over time. It facilitates targeted interventions in the following areas: finding purpose through meaningful activities and social connection, a proactive approach to seeking help and ensuring knowledge about accessing professional support, addressing negative or unhelpful assumptions and beliefs about civilians, validating and processing unresolved resentment and regrets about service, and addressing problematic rigidity and improving flexibility and adaptation. It provides the ability to assess these distinct factors that have been empirically associated with adjustment and successful reintegration. Finally, the measure is quick and simple to administer and score, with responders expected to take approximately 5 min to complete.

### Limitations & future directions

There are a number of limitations of this study, which may be addressed in future research. First, the validation process only included self-report methods. This potentially raises the likelihood of participant bias including social desirability and response sets. Given participants of this study were non-identifiable and the survey was administered online, it is unlikely that such bias had a meaningful impact on results, but the potential impact of self-selection bias cannot be determined from these data. In line with this, the response rate is unable to be determined as this research was conducted online with open recruitment and advertising through social media, which may have limited the representativeness of the sample. Second, there was considerable variability in responses and scores on the scale, which were associated with differences in adjustment as measured on other scales. Nonetheless, future validation research on the scale may benefit from incorporating additional objective or observable behavioural measures, such as utilization of health and social services. Third, longitudinal data was not collected and predictive validity not assessed. Future studies should consider a longitudinal approach, to enable evaluation of whether interpretation of test scores can predict outcomes of interest over time and assessment of the scale’s predictive validity and sensitivity to change over time. This study found that the five-factor structure was replicated in recently separated veterans samples (3 and 5 years post separation), providing initial indication of a robust factor structure irrespective of time since separation from the military. However, evaluating differences in profiles associated with time since separation in a larger sample may also be valuable, as well as investigation of factor invariance across gender, age, type of separation and time since separation for instance.

Future research on this measure may also include application and validation with other military cohorts globally including US, United Kingdom, Canada, and New Zealand, given the existing research, cultural similarities, and strong interest from these nations in improving transition and reintegration for their defence force personnel [9, 51, 52]. Finally, empirical development of a pre-separation version of the measure would be of use, allowing assessment of needs prior to permanently separating from the military. This would facilitate a proactive and ‘pre-habilitation’ approach to transition and reintegration – identifying at risk personnel and supporting skills building and reintegration training before transition and reintegration challenges arise. This version of the measure is currently in development by the present study research team. A test manual including test instructions, assessment procedure, scoring and interpretation guidelines will be publically available through the website: www.m-carm.org. Normative data will also be added to this website when further samples have been collected.

## Conclusion

The Military-Civilian Adjustment and Reintegration Measure (M-CARM) is the first measure of its kind designed to address the growing evidence of reintegration challenges for defence force personnel as they permanently transition out of the military into civilian life. The M-CARM specifically enables assessment of psychological adjustment and cultural reintegration following permanent separation from the military. Developed using both inductive and deductive methods, psychometric evaluation of the M-CARM demonstrated robust reliability and construct validity including convergent, divergent and discriminant validity as well as internal consistency and temporal stability. The hypothesized 21-item, 5-factor structure identified via EFA was verified by CFA using split random halves of the sample. The five factors include *Purpose and Connection*, *Help seeking*, *Beliefs about civilians*, *Resentment and Regret*, and *Regimentation*, with a higher order factor of *Adjustment and Reintegration*. This self-report measure can be completed in 5 min and has promising clinical practice and research application.

## Supplementary information

**Additional file 1.** Focus Group Questions. Qualitative Focus Group Questions - Health Professional Participants.

**Additional file 2.** Veteran Interview Questions. Qualitative Interview Questions - Veteran Participants.

**Additional file 3.** Partner Interview Questions. Qualitative Interview Questions - Partner of Veteran Participants.

**Additional file 4.** EFA 3 years separated. Exploratory Factor analysis of the M-CARM for participants who have been separated from Defence up to 3 years.

**Additional file 5.** EFA 5 years separated. Exploratory Factor analysis of the M-CARM for participants who have been separated from Defence up to 5 years.

## Data Availability

The data that support the findings of this study are available from Gallipoli Medical Research Foundation but restrictions apply to the availability of these data, which were used under license for the current study due to the regulations required of Defence Personnel, and so are not publicly available. Data are however available from the authors upon reasonable request and with permission of Australian Department of Defence and Veteran Affairs Human Research Ethics Committee.

## Abbreviations

ADF: Australian Defence Force
CFA: Confirmatory Factor Analysis
CFI: Comparative Fit Index
DASS-21: Depression Anxiety Stress Scale-21
EFA: Exploratory Factor Analysis
GMRF: Gallipoli Medical Research Foundation
PCL-5: PTSD Checklist for DSM-5
PTSD: posttraumatic stress disorder
M-CARM: Military-Civilian Adjustment and Reintegration Measure
M2C-Q: Military to Civilian Questionnaire
NDQ: Nightmare Distress Questionnaire
NFI: Normed Fit Index
RMSEA: Root Mean-Square Error of Approximation
TLI: Tucker-Lewis Fit Index
US: United States
WHOQOL-BREF: The World Health Organization Quality of Life Instrument
WRFIS: Walter Reed Functional Impairment Scale
