# Supplementary material for: Assessing psychological adjustment and cultural reintegration after military service: development and psychometric evaluation of the post-separation Military-Civilian Adjustment and Reintegration Measure (M-CARM)
Source: BMC Psychiatry. 2020 Nov 10;20:531. doi: 10.1186/s12888-020-02936-y (PMC7654614; doi:10.1186/s12888-020-02936-y)
Supplement: Supplementary file 1 — Additional file 1. Focus Group Questions. Qualitative Focus Group Questions - Health Professional Participants. [file 12888_2020_2936_MOESM1_ESM.docx]

Name: Additional file 1

Title: Focus Group Questions

Description: Qualitative Focus Group Questions - Health Professional Participants

Author: Dr Madeline Romaniuk, Gallipoli Medical Research Foundation

Section 1: Orientation

This document outlines focus group questions for use with participants of the VMH-01 research study. This should be considered as a guide rather than a prescriptive protocol in line with qualitative research, which emphasises an un-structured, open-ended and participant-centred interview style. The interviewer should focus on building rapport and facilitating a conversational focus group style that is led by the participants – allowing them to fully explain and elaborate on their experiences. The questions below can be altered at the discretion of the interviewer within and between focus group session/data collection in keeping with qualitative methodology.

Section 2: Questions

Q1. Describe military culture as you see it based on your experiences with the veteran and currently serving military population.

Q2. In your work, do you see ex-military patients (who may have been out for a long time) that still embody any parts of this culture? If yes, please describe the key parts of the culture they embody.

Q3. What are the differences between military and civilian culture? Describe from the perceptive of military personnel – the divide between military vs civilian culture – as they report/experience it.

Q4. What are your specific observations of ex-military personnel who have difficulty fitting in to civilian life? Can you provide specific (but unidentifiable) examples? i.e. How can you tell when patients have not been able to adjust? Behaviours, attitudes, assumptions, biases etc.

Q5. What are the key factors that prevent veterans from psychologically adjusting to civilian life?

Q6. What are the differences between veterans that adjust well to civilian life versus those that do not? i.e. positive predictive factors to successful adjustment/reintegration to civilian life.

Q7. What treatment/intervention would help address cultural reintegration? Are you using any intervention
