# Supplementary material for: Assessing psychological adjustment and cultural reintegration after military service: development and psychometric evaluation of the post-separation Military-Civilian Adjustment and Reintegration Measure (M-CARM)
Source: BMC Psychiatry. 2020 Nov 10;20:531. doi: 10.1186/s12888-020-02936-y (PMC7654614; doi:10.1186/s12888-020-02936-y)
Supplement: Supplementary file 2 — Additional file 2. Veteran Interview Questions. Qualitative Interview Questions - Veteran Participants. [file 12888_2020_2936_MOESM2_ESM.docx]

Name: Additional file 2

Title: Veteran Interview Questions

Description: Qualitative Interview Questions - Veteran Participants

Author: Dr Madeline Romaniuk, Gallipoli Medical Research Foundation

Section 1: Orientation

This document outlines a guide for conducting interviews with participants of the VMH-01 research study. This should be considered as a guide rather than a prescriptive protocol in line with qualitative research, which emphasises an un-structured, open-ended and participant-centred interview style. The interviewer should focus on building rapport and facilitating a conversational interview style that is led by the participant – allowing them to fully explain and elaborate on their experiences. The questions below can be altered at the discretion of the interviewer within and between interviews/data collection in keeping with qualitative methodology.

Section 2: Questions

Q1. Tell me why you decided to join the Military? i.e. What appealed to you about joining?

Q2. What were you doing before you joined the Military?

Q3. What was your experience like in the Military?

Q4. What did you like and dislike about it?

Q5. What’s your interpretation of the word ‘culture’?

Q6. Describe military culture as you see it.

Q7. What does being a part of that culture mean to you?

Q8. Do you still feel like you are part of that culture?

If yes, does it feel different now as an ex- serving member?

If no, what does not being a part of that culture feel like?

Q9. How would describe civilian culture as you see it?

Q10. What do you see are the differences between military and civilian culture?

Q11. What was it like leaving the military? (Practically, emotionally, psychologically). Describe the process of transition.

Q12. Have you experienced any difficulties fitting in to civilian life again? Please describe.

Q13. When do you notice these difficulties most? (what settings/circumstances)

Q14. What has been helpful in managing these difficulties? (Personality/social factors, support services)

Q15. Do you think you have mentally adapted to civilian life again?

If yes, why do you think that is? How were you able to do that?

If no, why do you think that is?

Q16. What other things have helped you (or prevented you from) adapting back to civilian life?

Q17. What advice would you give to a mate who was about to leave the Military? How would you prepare them?

Q18. What would have made the process easier/better for you?
