# Supplementary material for: Assessing psychological adjustment and cultural reintegration after military service: development and psychometric evaluation of the post-separation Military-Civilian Adjustment and Reintegration Measure (M-CARM)
Source: BMC Psychiatry. 2020 Nov 10;20:531. doi: 10.1186/s12888-020-02936-y (PMC7654614; doi:10.1186/s12888-020-02936-y)
Supplement: Supplementary file 3 — Additional file 3. Partner Interview Questions. Qualitative Interview Questions - Partner of Veteran Participants. [file 12888_2020_2936_MOESM3_ESM.docx]

Name: Additional file 3

Title: Partner Interview Questions

Description: Qualitative Interview Questions - Partner Participants

Author: Dr Madeline Romaniuk, Gallipoli Medical Research Foundation

Section 1: Orientation

This document outlines a guide for conducting interviews with participants of the VMH-01 research study. This should be considered as a guide rather than a prescriptive protocol in line with qualitative research, which emphasises an un-structured, open-ended and participant-centred interview style. The interviewer should focus on building rapport and facilitating a conversational interview style that is led by the participant – allowing them to fully explain and elaborate on their experiences. The questions below can be altered at the discretion of the interviewer within and between interviews/data collection in keeping with qualitative methodology.

Section 2: Questions

Q1. What was the process of leaving the military like for you and your partner? Describe the experience.

Q2. Describe military culture as you see it based on your experiences with your partner.

Q3. Does your partner still embody any parts of this culture? If yes, please describe.

Q4. What do you see are the differences between military and civilian culture?

Q5. Has your partner experienced any difficulties fitting in to civilian life again? Please describe.

Q6. When do you notice these difficulties most? (what settings/circumstances)?

Q7. What has been helpful in managing these difficulties? (Personality/social factors, support services)

Q8. Do you think they have mentally adapted to civilian life again?

If yes – How do you think they were able to do that?

If no – what prompts you to think that?

Q9. What other things have helped them adapt back to civilian life?

Q10. What other things have prevented them adapting to civilian life?

Q11. What would have made the process easier/better for you and your partner?
