# Supplementary material for: Assessing psychological adjustment and cultural reintegration after military service: development and psychometric evaluation of the post-separation Military-Civilian Adjustment and Reintegration Measure (M-CARM)
Source: BMC Psychiatry. 2020 Nov 10;20:531. doi: 10.1186/s12888-020-02936-y (PMC7654614; doi:10.1186/s12888-020-02936-y)
Supplement: Supplementary file 4 — Additional file 4. EFA 3 years separated. Exploratory Factor analysis of the M-CARM for participants who have been separated from Defence up to 3 years. [file 12888_2020_2936_MOESM4_ESM.docx]

Name: Additional file 4

Title: EFA 3 years separated

Description: Exploratory Factor analysis of the M-CARM for participants who have been separated from Defence up to 3 years.

**Table 1**

*Oblique rotated five-factor solution for the 21-Item Military-Civilian Adjustment and Reintegration Measure in those who separated up to 3 years prior (n = 204)*

| Item | Factors | | | | |
| --- | --- | --- | --- | --- | --- |
|  | 1 | 2 | 3 | 4 | 5 |
| 43. I have things that give me a sense of purpose, outside of paid employment. | .847* | -.037 | .001 | -.028 | -.022 |
| 30. I have interests and hobbies that are enjoyable or meaningful. | .819* | -.005 | .019 | -.006 | -.010 |
| 46. I have a sense of purpose. | .685* | .036 | -.135 | -.030 | .113 |
| 14. Outside of the military, I have found people that I connect with through shared interests or beliefs. | .639* | .089 | .040 | .048 | .024 |
| 38. I am fulfilled. | .633* | .044 | -.146 | .110 | .088 |
| 47. I feel I don’t belong anywhere. | .608* | .012 | -.192 | -.008 | .149 |
| 34. I would ask for help if I needed it. | .221 | .876* | .180 | .032 | .003 |
| 21. I would never seek help from a mental health professional. | -.193 | .643* | .180 | .032 | .003 |
| 40. I find it difficult to ask for help if I’m struggling. | .031 | .521* | -.017 | .199 | -.030 |
| 1. I know how to access professional support for my health. | .044 | .442* | -.100 | .003 | .022 |
| 26. Despite all my experience in the military, I am undervalued by civilians. | .107 | .077 | -.760* | .006 | -.071 |
| 17. I don’t think society puts much value on military service and experience. | -.016 | -.014 | -.673* | -.061 | .143 |
| 36. Civilians seem to be concerned with trivial matters. | .041 | -.007 | -.542* | .108 | -.087 |
| 15. Civilians are disrespectful and rude. | .037 | .058 | -.532* | .054 | .135 |
| 42. I am more regimented than flexible. | -.109 | .059 | .012 | .798* | .128 |
| 32. I find it difficult to change once I have a set routine. | .022 | .014 | -.062 | .680* | -.022 |
| 5. I am a flexible person and I don’t mind changing to suit others when required. | .239 | -.021 | .023 | .513* | -.015 |
| 3. Some of my military habits cause problems for me. | -.023 | -.026 | -.226 | .468* | .118 |
| 12. I’m angry about the way I was treated during my service. | -.040 | .045 | -.009 | -.041 | .880* |
| 20. The military broke me and then kicked me out. | .076 | .005 | -.007 | .113 | .585* |
| 41. I have a lot of regrets about my service. | .225 | -.044 | -.001 | .108 | .482* |
| Variance explained (%) | 30.60 | 7.59 | 6.19 | 4.46 | 3.84 |
| Eigenvalues | 6.86 | 2.07 | 1.73 | 1.39 | 1.24 |

*Note.* *Loadings > 0.40

**Table 2**

*Demographic Characteristics (0-3 years since discharge)*

| Demographic variables | Full sample  (*n* = 204) |
| --- | --- |
| Age, *M* (*SD*), range | 41.43 (10.37), 21-66 |
| Gender, % (*n*) |  |
| Female | 78.92 (43) |
| Male | 21.08 (161) |
| Did not disclose | 0.00 (0) |
| Marital status |  |
| Single | 26.47 (54) |
| Married | 52.45 (107) |
| Partner/De facto | 21.08 (43) |
| Highest level of education |  |
| No education | 0.49 (1) |
| Secondary | 59.80 (122) |
| University | 39.71 (81) |
| Service Type, % (*n*) |  |
| Army | 63.24 (129) |
| Navy | 17.16 (35) |
| Air Force | 16.18 (33) |
| More than one | 3.43 (7) |
| Years of service, *M* (*SD*) range | 17.84 (10.17), 1-46 |
| Years since separation | 1.62 (1.04), 0-3 |
| Deployed to combat zone, % (*n*) | 70.59 (144) |
| Medically discharged, % (*n*) | 47.55 (97) |
| Self-report psychological condition, % (*n*) |  |
| Yes | 59.31 (121) |
| No | 33.33 (68) |
| Unsure | 7.35 (15) |
| Provisional PTSD diagnosis, % (*n*) | 43.63 (89) |
| Received psychological treatment, % (*n*) |  |
| Yes | 61.76 (126) |
| No | 3.43 (7) |
| Unsure | 1.47 (3) |
| Not applicable | 33.33 (68) |
| Employment Status, % (*n*) |  |
| Full-time | 39.71 (81) |
| Part-time/Casual | 13.24 (27) |
| Retired/pension | 33.33 (68) |
| Unemployed | 9.31 (19) |
| Other (e.g. student, volunteer) | 4.41 (9) |
| Submitted Veteran Affairs claim, % (*n*) | 73.02 (151) |
