# Supplementary material for: Assessing psychological adjustment and cultural reintegration after military service: development and psychometric evaluation of the post-separation Military-Civilian Adjustment and Reintegration Measure (M-CARM)
Source: BMC Psychiatry. 2020 Nov 10;20:531. doi: 10.1186/s12888-020-02936-y (PMC7654614; doi:10.1186/s12888-020-02936-y)
Supplement: Supplementary file 5 — Additional file 5. EFA 5 years separated. Exploratory Factor analysis of the M-CARM for participants who have been separated from Defence up to 5 years. [file 12888_2020_2936_MOESM5_ESM.docx]

Name: Additional file 5

Title: EFA 5 years separated

Description: Exploratory Factor analysis of the M-CARM for participants who have been separated from Defence up to 5 years.

**Table 1**

*Oblique rotated five-factor solution for the 21-Item Military-Civilian Adjustment and Reintegration Measure in those who separated up to 5 years prior (n = 301)*

| Item | Factors | | | | |
| --- | --- | --- | --- | --- | --- |
|  | 1 | 2 | 3 | 4 | 5 |
| 43. I have things that give me a sense of purpose, outside of paid employment. | .824* | .018 | -.005 | -.059 | .010 |
| 30. I have interests and hobbies that are enjoyable or meaningful. | .805* | .008 | -.056 | -.028 | .031 |
| 46. I have a sense of purpose. | .727* | -.009 | .160 | -.027 | .066 |
| 38. I am fulfilled. | .668* | .019 | .140 | .147 | .022 |
| 47. I feel I don’t belong anywhere. | .657* | .010 | .160 | .082 | .074 |
| 14. Outside of the military, I have found people that I connect with through shared interests or beliefs. | .613* | .077 | -.007 | .052 | -.001 |
| 34. I would ask for help if I needed it. | .205 | .825* | -.167 | .078 | -.030 |
| 21. I would never seek help from a mental health professional. | -.146 | .703* | .102 | -.112 | -.021 |
| 40. I find it difficult to ask for help if I’m struggling. | .098 | .543* | -.016 | .173 | .028 |
| 1. I know how to access professional support for my health. | -.008 | .507* | .053 | -.030 | .054 |
| 36. Civilians seem to be concerned with trivial matters. | .050 | .000 | .530* | .175 | -.161 |
| 26. Despite all my experience in the military, I am undervalued by civilians. | .153 | .052 | .663* | .067 | -.056 |
| 15. Civilians are disrespectful and rude. | .072 | .060 | .591* | .037 | .086 |
| 17. I don’t think society puts much value on military service and experience. | -.034 | -.008 | .679* | -.098 | .175 |
| 42. I am more regimented than flexible. | -.139 | .051 | .019 | .820* | .081 |
| 32. I find it difficult to change once I have a set routine. | .073 | -.006 | .020 | .664* | .023 |
| 5. I am a flexible person and I don’t mind changing to suit others when required. | .232 | -.049 | -.028 | .509* | .008 |
| 3. Some of my military habits cause problems for me. | .000 | .022 | .148 | .520* | .081 |
| 12. I’m angry about the way I was treated during my service. | -.024 | .069 | .001 | -.001 | .834* |
| 20. The military broke me and then kicked me out. | .040 | -.013 | .034 | .191 | .544* |
| 41. I have a lot of regrets about my service. | .245 | -.006 | .025 | .043 | .440* |
| Variance explained (%) | 31.03 | 7.34 | 5.72 | 4.57 | 3.93 |
| Eigenvalues | 6.96 | 2.02 | 1.66 | 1.42 | 1.28 |

*Note.* *Loadings > 0.40

**Table 2**

*Demographic Characteristics (0-5 years since discharge)*

| Demographic variables | 0-5 Years Discharged Sample  (*n* = 301) |
| --- | --- |
| Age, *M* (*SD*), range | 41.33 (10.05), 21-66 |
| Gender, % (*n*) |  |
| Female | 19.27 (58) |
| Male | 80.73 (243) |
| Did not disclose | 0.00 (0) |
| Marital status |  |
| Single | 23.26 (70) |
| Married | 55.48 (167) |
| Partner/De facto | 21.26 (64) |
| Highest level of education |  |
| No education | 0.66 (2) |
| Secondary | 59.47 (179) |
| University | 39.87 (120) |
| Service Type, % (*n*) |  |
| Army | 62.79 (189) |
| Navy | 16.28 (49) |
| Air Force | 17.28 (52) |
| More than one | 3.65 (11) |
| Years of service, *M* (*SD*) range | 17.04 (9.93), 1-46 |
| Years since separation | 2.55 (1.62), 0-5 |
| Deployed to combat zone, % (*n*) | 68.44 (206) |
| Medically discharged, % (*n*) | 46.51 (140) |
| Self-report psychological condition, % (*n*) |  |
| Yes | 60.47 (182) |
| No | 32.22 (97) |
| Unsure | 7.31 (22) |
| Provisional PTSD diagnosis, % (*n*) | 33.19 (133) |
| Received psychological treatment, % (*n*) |  |
| Yes | 62.46 (188) |
| No | 3.65 (11) |
| Unsure | 1.67 (5) |
| Not applicable | 32.22 (97) |
| Employment Status, % (*n*) |  |
| Full-time | 41.86 (126) |
| Part-time/Casual | 12.62 (38) |
| Retired/pension | 32.22 (97) |
| Unemployed | 9.63 (29) |
| Other (e.g. student, volunteer) | 3.65 (11) |
| Submitted Veteran Affairs claim, % (*n*) | 73.42 (221) |
